# Effects of traditional Chinese herb hot compress combined with therapeutic exercise on pain, proprioception, and functional performance among older adults with knee osteoarthritis: A randomized controlled trial

**DOI:** 10.3389/fphys.2022.1070754

**Published:** 2022-12-14

**Authors:** Jingwen Wang, Wei Liu, Haitao Fu

**Affiliations:** ^1^ College of Sports and Health, Shandong Sport University, Jinan, China; ^2^ Department of Rehabilitation, Neck Shoulder Back and Leg Pain Hospital, Shandong First Medical University, Jinan, China; ^3^ Athletic Training Division, Shandong Sport University, Jinan, China

**Keywords:** osteoarthritis, pain, proprioception, functional performance, Chinese herbs, therapeutic exercise

## Abstract

**Background:** Knee osteoarthritis (KOA) is one of the most common chronic progressive diseases with degenerative destruction of articular cartilage and bone, leading to knee pain, impaired proprioception, and reduced functional performance. This study was to investigate the effects of an 8-week Traditional Chinese herb hot compress (TCHHC) combined with therapeutic exercise (TE) on pain, proprioception, and functional performance among older adults with KOA.

**Methods:** Twenty-seven older adults with KOA were recruited and randomly assigned to the TCHHC + TE or TE groups. Thirteen participants received TCHHC + TE, and fourteen received TE. At pre- (week 0) and post-intervention (week 9), their pain, joint proprioception, and functional performance were measured. Two-way ANOVA with repeated measures was adopted to analyze the data.

**Results:** Compared with week 0, the pain score, proprioception thresholds of knee extension and ankle plantarflexion, and the times of TUG and 20-m walk tests decreased more significantly in the TCHHC + TE group than in the TE group at week 9.

**Conclusion:** Compared with TE, the 8-week TCHHC + TE was superior in relieving pain, recovering proprioception, and improving functional performance among older adults with KOA. It is recommended that TCHHC should be adopted prior to TE to enhance the effects of KOA rehabilitation.

## 1 Introduction

Knee osteoarthritis (KOA) is one of the most common chronic progressive diseases, with a prevalence of 30%–40% among older adults over 65 years of age, posing a serious health risk (Helmick et al., 2008). KOA is associated with degenerative destruction of articular cartilage and bone, leading to knee pain (Glyn-Jones et al., 2015), impaired proprioception (Segal et al., 2010), and reduced functional performance (Hawker, 2019).

Pain is the predominant and most common clinical symptom of KOA, which was believed to be caused by the uneven load at the knee and the increased pressure on the tibiofemoral joint (Beaupré et al., 2000). Pain has been commonly assessed by the Western Ontario and McMasters Osteoarthritis Index (WOMAC) (Sisante et al., 2020). Proprioception is closely related to the developmental process of KOA (van der Esch et al., 2007), and the impairment of proprioception of the knee and ankle joints led to decreased lower extremity joint balance and coordination among older adults with KOA (Hassan et al., 2001; Knoop et al., 2011). Although KOA affects mostly the knee joint, ankle proprioception is closely related to balance control (Han et al., 2015). Compared with their healthy counterparts, older adults with KOA have worse functional performance, which affects their ability of daily activities and increases the risk of falls (Anderson et al., 2019). Timed up & Go (TUG) (Podsiadlo and Richardson, 1991; Dobson et al., 2012) and 20-m walk tests (Motyl et al., 2013) have been used effectively to quantify functional performance among older adults with KOA.

Older adults with KOA typically receive surgical, pharmacological, and non-pharmacological treatments (Brophy and Fillingham, 2022). Surgery is generally used in the late-stage and can improve symptoms of KOA (Xue et al., 2022), however, with a high cost (Serikova-Esengeldina et al., 2022); Non-steroidal antiinflammatory, opioids, or nutritional drugs are usually used in pharmacological treatments (Persson et al., 2018), and side effects on the liver and kidneys have been reported (Zhang et al., 2019b). Non-pharmacological treatments have been recommended as the primary treatments for KOA (Brophy and Fillingham, 2022). Among them, therapeutic exercise (TE), such as resistance training or joint mobilization, has the potential effects to relieve pain (Juhl et al., 2014), recover proprioception (Lai et al., 2018), and improve functional performance (Fransen et al., 2002). Resistance training improves muscle strength and functional performance (de Rooij et al., 2017). Joint mobilization balances the load at the knee and relieves pain (Chen H. et al., 2019), as well as improves proprioception by enhancing sensory input to the central nervous system about postural movements (Saunders et al., 2005). However, a previous meta-analysis study showed that the effect size of TE remained small to moderate (Fransen et al., 2015). Therefore, there is room to improve its effectiveness on KOA.

Traditional Chinese herb hot compress (TCHHC) has been widely used in the treatment of chronic diseases such as KOA (Yuan et al., 2015). It fully synergizes the warming effect, which has been shown to slightly improve pain, functional performance and quality of life in older adults with KOA (Aciksoz et al., 2017), and the pharmacological effect, which reduces inflammation for pain relief by promoting synovial fluid flow and reducing the release of inflammatory substances into the synovial fluid (Wang et al., 2010). Combined with the above-mentioned effects of resistance training and joint mobilization, TCHHC + TE has the potential to relieve pain, improve proprioception, and functional performance.

To the best of our knowledge, no studies have explored the effects of TCHHC + TE on pain relief, proprioception recovery, and functional performance improvement among older adults with KOA. It was hypothesized that compared with TE, TCHHC + TE was superior in relieving pain, recovering proprioception, and reducing the times of TUG and 20-m walk tests.

## 2 Material and methods

### 2.1 Sample size estimates

The sample size was estimated by An *a priori* power analysis (G*Power Version 3.1). Based on a previous report compared the pain score (interaction <0.001, η2_p_ = 0.638) and proprioception (interaction = 0.006, η2_p_ = 0.267) among older adults with KOA before and after a TE therapy or health lecture series (Shen et al., 2021). By setting the significance level to 0.05 and the statistical power to 80%, the minimum total sample size of this study should be 6 (calculated by pain score) and 26 (calculated by proprioception), respectively.

### 2.2 Participants

All participants were recruited and all the data were collected in the Neck, Shoulder, Back, and Leg Pain Hospital, Jinan, China, from June 2020 to November 2021. The inclusion criteria included the following: 1) 65 years old or older; 2) at least one knee was diagnosed with KOA according to the clinical criteria of the American College of Rheumatology; 3) a radiographic grade of 2 or higher by the Kellgren/Lawrence scale (K/L). The exclusion criteria included the following: 1) had a neurodegenerative disease or neurosensory disorder affecting the knee other than the KOA; 2) had a traumatic injury of the lower extremity joint in the past 3 months; 3) had planned for a total knee replacement in the following months; 4) had a history of allergies to Traditional Chinese herbs; 5) had received other KOA treatments, including steroids, intra-articular injection or other analgesic drugs within previous 3 months.

Thirty-four participants who met the above criteria were assigned to the TCHHC + TE or the TE groups at a ratio of 1:1 by computer-generated randomization procedure. Allocation concealment was ensured because allocation information was protected in opaque sealed envelopes and kept by investigators involved in participant recruitment. This study was single-blinded, with the investigators were blinded and participants knew the randomized assignments.

**FIGURE 1:**
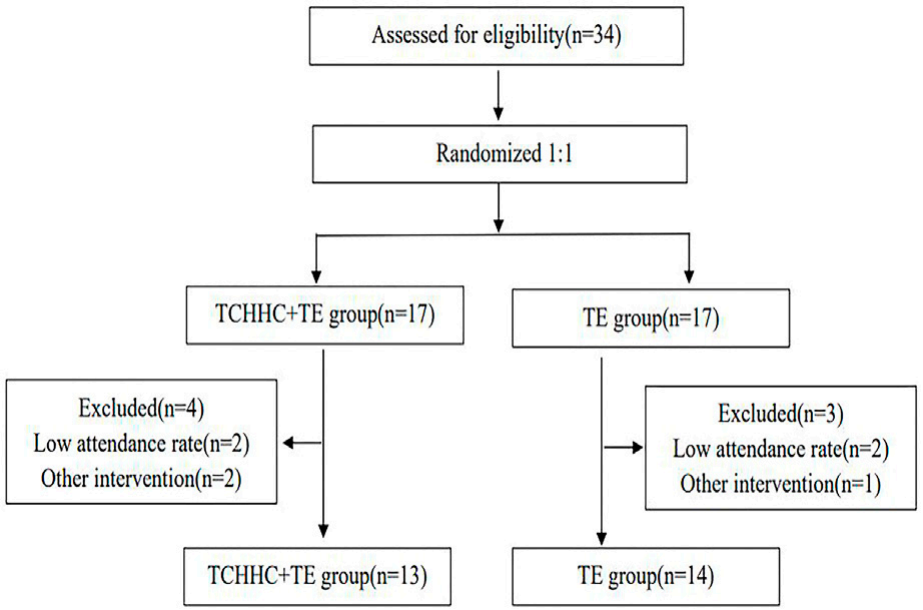
Chart flow.

The participants in the TCHHC + TE group received TCHHC + TE, and those in the TE group received TE for 8 weeks. The participants were excluded with below 80% attendance rate (Song et al., 2020). The attendance rate is calculated by dividing the number of sessions completed by each participant by the total number of sessions. In the TCHHC + TE group, two participants were excluded due to their lower attendance rate (73% and 70%), and two were excluded due to co-intervention. In the TE group, two were excluded due to their lower attendance rate (74% and 72%), and one was excluded due to co-intervention. The reasons for failing to attend were transportation difficulties, bad weather, and family commitments. Co-intervention including steroids, intra-articular injections or other pain medications. Final analyses were conducted among thirteen participants in the TCHHC + TE group and fourteen in the TE group (Figure 1).

All participants were required to sign informed consent before participation. The project was approved by the Ethics Committee of Neck, Shoulder, Back, and Leg Pain Hospital (2021009) and conformed to the guidelines of the Declaration of Helsinki, and was registered in the Chinese Clinical Trial Registry with a registration number of ChiCTR2100052450.

### 2.3 Interventions

Both groups received three sessions (Tuesday, Thursday, and Saturday) of the intervention per week for 8 weeks. Participants were tested successively on Monday of the ninth week. In each session, the participants in the TCHHC + TE group received 30 min of TCHHC, 30 min of joint mobilization, and 30 min of resistance training. The TE group received 30 min of joint mobilization, 30 min of resistance training, and 30 min of health lecture series.

**FIGURE 2:**
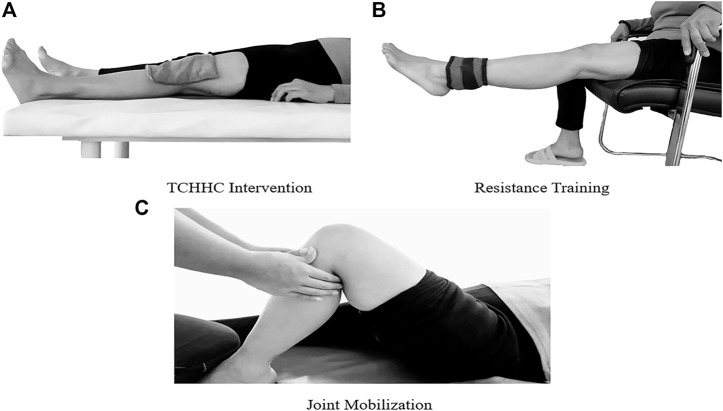
Illustrations of TCHHC **(A)**, resistance training **(B)**, and joint mobilization **(C)**.

The dosage of the herbs was confirmed by the Apoplectic Joint Disease Pulse Syndrome and Treatment, Guizhi Shaoyao Zhimu Decoction, prescribed by Zhang Zhongjing, a famous Chinese medical practitioner of the Eastern Han Dynasty (from about 150 to 154 to about 215 to 219, A.D.) (Daily et al., 2017) and provided by the Neck, Shoulder, Back, and Leg Pain Hospital. The types and dosages of hot compress herbs are shown in Table1. The herbs were ground into 0.2 mm power in the hospital by the Dade medicine machine (model DF-20, Wenling Linda Machinery Co., Ltd, China), and mixed in a cloth bag of about 15 cm length*15 cm width after heating in a microwave oven for 40 s to make the temperature of the medicine bag reach 40–42°C, measured using the Raytek MiniTemp™ non-contact infrared thermometer (Raytek, United States). After cleaning the skin with saline gauze, the bag was quickly applied to the knee joint for about 30 min (Figure 2A).

**TABLE 1:** Components of Traditional Chinese herbs.

Components	Amount (g)	Components	Amount (g)
Cassia twig	30	White mustard seed	30
Paeony root	20	Sichuan ox knee	25
Myrrh	20	Mulberry parasitic	25
Ephedra root	20	Parsnip	20
Atractylodes macrocephala	20	Hot dog ridge	30
Frankincense	20	Antler gum	30
Prepared aconite	25	Anemarrhena asphodeloides	20
Prepared Sichuan black	25		

The TE intervention included two parts, namely, resistance training (static and dynamic) and joint mobilization. During static resistance training, the participants lay in a supine position, contracted their quadriceps to keep their heels slightly off the bed for 10 s, and relaxed for 10 s, repeating about 50 reps per session. During dynamic resistance training, the participant sat in a training chair and flexed and extended the knee at approximately 90° to 170° with a weight adjustable bag (between 9.8 and 15N depending on the participant’s knee recovery) strapped to the ankle to increase knee extension resistance for about 100 reps per session (Figure 2B). The joint mobilization was based on Maitland’s method in Australia (Courtney et al., 2010). The participants lay in a supine position and received joint mobilization conducted by physical therapists about 8 times per session. The joint mobilization technique involved the following: medial and lateral sliding of the patella, and up and down, anterior and posterior sliding of the tibiofibular joint, anterior and posterior sliding of the tibiofemoral joint, and long-axis traction (Figure 2C).

All participants in the TE group participated in the health lecture series for 8 weeks, three sessions per week. One session of health lecture lasted for 30 min, including knowledge about KOA, awareness of chronic diseases, psychological health education, nutritional meals, scientific exercise, and exchange of experience. The format of the lecture was to watch selected TV programs or read related magazines.

### 2.4 Testing protocol

#### 2.4.1 Pain

The pain score of the more affected lower extremity was assessed by five pain items of the WOMAC before and after the 8-week intervention, and its validity and reliability have been demonstrated (Bellamy et al., 1988). In each item, 0 points represented “no pain,” whereas 10 points represented “the worst pain possible”. Higher scores indicate more severe pain.

#### 2.4.2 Proprioceptive

The proprioceptive thresholds of knee flexion/extension, ankle dorsi/plantarflexion, and inversion/eversion of the more affected lower extremity were measured by proprioception devices (Sunny, AP-II, China). The test showed a good two-time-point intraclass correlation coefficient ((ICC = 0.737–0.935) (Sun et al., 2015). The proprioception devices collected the minimum angular motion that the patient was able to detect during knee flexion/extension, ankle dorsi/plantarflexion, and inversion/eversion. The devices consist of a platform that can rotate within the frontal and sagittal planes. The platform is driven by 2 electric motors at an angular velocity of 0.4°/s. An electronic goniometer in the devices recorded the angular displacement of the platform. Each participant was seated on a height-adjustable chair with their foot placed on the platform. During the ankle proprioception test, the knee and hip joints were flexed at 90°, and the leg was perpendicular to the surface of the platform when the platform was placed in a horizontal position. During the knee proprioception test, the lateral axis of the instrumentation was parallel with the mediolateral axis of the knee joint. The hip and knee joints were each positioned at 90°, whereas the ankle joint was at the neutral position. Approximately 50% subject’s lower extremity weight was rested on the platform by using the thigh cuff suspension system to control unwanted sensory cues from the contact between the platform and the plantar surface of the foot. The participant sat with their eyes closed and wore headphones with music playing to eliminate potential environmental visual and auditory stimulation. The participant was instructed to concentrate on their foot and to press the hand switch to stop the movement of the platform when they could sense motion followed by identification of the rotation direction. The motor was operated to rotate with a random time interval ranging from 2 s to 10 s after an indication to start a trial. At least five trials were performed for each direction to reduce random measurement errors (Song et al., 2021).

#### 2.4.3 TUG test

The TUG test showed good reliability between raters (ICC = 0.99) and within the same raters on two consecutive tests (ICC = 0.99) (Podsiadlo and Richardson, 1991). The participants were seated in a 46 cm high seat. When the assistant gave the “go” command, the participants stood up and moved forward a 3-m distance as fast as possible, then turned around and sat back down. The time was taken from when they left the chair until they returned to the chair, in seconds. Shorter time represents better functional performance. Three successful trials were collected.

#### 2.4.4 20-m walk test

The 20-m walk test showed good two-time-point reliability (Spearman r = 0.94–0.99) (Motyl et al., 2013). The participants walked as fast as possible within a 20-m distance. Shorter time represents better functional performance. Three successful trials were collected.

### 2.5 Statistical analysis

All variables were statistically analyzed using SPSS 26.0 (IBM SPSS, Armonk, NY, United States). The normality of all outcome variables was tested using Shapiro-Wilk tests. Two-way (group by time) ANOVAs with repeated measures were used to test the differences in dependent variables before and after the intervention. If significant interactions were detected, the stratified t-tests with the Bonferroni adjustment were performed. If there is no interaction, pre-and post-tests were compared by combining the two groups. Partial eta squared (η2_p_) was used to represent the effect size of the two-way ANOVA’s main effects and interactions. The thresholds for η2_p_ were as follows: 0.01–0.06 for small, 0.06–0.14 for moderate, and>0.14 for large (Ledolter and Kardon, 2020). Cohen’s *d* was used to represent the effect size of *post hoc* pairwise comparisons (Cohen, 1988). The thresholds for Cohen’s *d* were as follows: <0.20 for trivial, 0.21–0.50 for small, 0.51–0.80 for medium, and >0.81 for large (J, 1988). The significance level was set at 0.05 (Ledolter and Kardon, 2020).

## 3 Results

No side effects were observed during the intervention, e.g., pain exacerbation, allergic reaction, redness, or heat burn due to the hot compress on the superficial joints. The Shapiro-Wilk test confirmed that all dependent variables were normally distributed. Independent t-tests showed no significant differences in age (*p* = 0.794), weight (*p* = 0.962), height (*p* = 0.467), body mass index (*p* = 0.841), and leg length (*p* = 0.639) between the two groups (Table 2).

**TABLE 2:** Demographic characteristics of practitioners.

	TCHHC + TE (*n* = 13)	TE (*n* = 14)	*p*-value
More affected leg	5R, 8L	6R, 8L	--
K/L	4 II, 7 III, 2 IV	4 II, 8 III, 2 IV	--
Age (y)	72.0 ± 5.4	72.6 ± 5.9	0.794
Weight (kg)	69.9 ± 5.2	70.0 ± 7.7	0.962
Height (cm)	162.2 ± 4.1	163.6 ± 5.7	0.467
BMI (kg/m^2^)	26.7 ± 2.2	26.5 ± 2.5	0.841
Leg length (cm)	82.3 ± 3.1	81.7 ± 3.4	0.639

The pain score was presented in Table 3. Significant interactions were detected in 2 items (walk on the ground: *p* = 0.009, η2_p_ = 0.245 and go up or down stairs: *p* = 0.006, η2_p_ = 0.266) of the WOMAC scores, and the total score (*p* = 0.047, η2_p_ = 0.148). At week 9, the go up or down stair (*p* = 0.002, *d* = 1.327) and total (*p* = 0.035, *d* = 0.798) scores were lower in the TCHHC + TE group compared with those in the TE group. Compared to week 0, the sleep at night (*p* < 0.001, η2_p_ = 0.395), sit or lying (*p* = 0.001, η2_p_ = 0.364), and stand straight (*p* < 0.001, η2_p_ = 0.424) scores were lower at week 9 in both groups.

**TABLE 3:** Pain score of the more affected at weeks 0 and 9.

Pain score		TCHHC + TE (*n* = 13)	TE (*n* = 14)	Time		Group		Time^a^ group		Post hoc	
				p	η^2^_p_	p	η^2^_p_	p	η^2^_p_	p	*d*
Walk on the ground	Week 0	5.08 ± 2.25	4.95 ± 1.38	--	--	--	--	0.009	0.245	^a^ ^1^ < 0.001,^a^ ^2^ < 0.001	^a^ ^1^ = 1.605,^a^ ^2^ = 1.874
	Week 9	1.85 ± 1.63^a^ ^1^	2.50 ± 1.22^a^ ^2^							--	--
Go up or down stairs	Week 0	6.62 ± 2.22	6.61 ± 1.75	--	--	--	--	0.006	0.266	^a^ ^1^ < 0.001,^a^ ^2^ < 0.001	^a^ ^1^ = 2.316,^a^ ^2^ = 1.822
	Week 9	2.15 ± 1.28^a^ ^1^ ^b^	3.79 ± 1.19^a^ ^2^							^b^ = 0.002	^b^ = 1.327
Sleep at night	Week 0	4.23 ± 2.65	4.36 ± 2.27	<0.001	0.395	0.596	0.011	0.302	0.043	--	--
	Week 9	1.31 ± 1.25	1.64 ± 1.40							--	--
Sit or lying	Week 0	2.85 ± 1.95	2.64 ± 1.28	0.001	0.364	0.835	0.002	0.745	0.004	--	--
	Week 9	1.23 ± 0.93	1.29 ± 1.14							--	--
Stand straight	Week 0	2.62 ± 1.80	2.92 ± 1.77	<0.001	0.424	0.059	0.135	0.257	0.051	--	--
	Week 9	1.38 ± 0.96	1.79 ± 1.37							--	--
Total score	Week 0	22.38 ± 7.43	20.35 ± 6.07	--	--	--	--	0.047	0.148	^a^ ^1^ < 0.001,^a^ ^2^ < 0.001	^a^ ^1^ = 2.246,^a^ ^2^ = 1.760
	Week 9	7.92 ± 3.92^a^ ^1^ ^b^	11.00 ± 3.80^a^ ^2^							^b^ = 0.035	^b^ = 0.798

^a^ Denotes significant different between weeks 0 and 9 in each group.

^b^ Denotes significant difference between the two groups at week 9.

TCHHC: traditional chinese herb hot compress.

TE: therapeutic exercise.

The proprioceptive thresholds were presented in Table 4. Significant interactions were detected in proprioception thresholds of knee extension (*p* = 0.018, η2_p_ = 0.203) and ankle plantarflexion (*p* = 0.010, η2_p_ = 0.235). Post hoc comparisons showed that compared with week 0, the proprioception thresholds of the knee extension and ankle plantarflexion of both groups (TCHHC + TE: *p* < 0.001, *d* = 1.870, *p* < 0.001, *d* = 2.434; TE: *p* = 0.007, *d* = 0.927; *p* < 0.001, *d* = 1.352) decrease at week 9. The proprioception threshold of the knee extension was lower in the TCHHC + TE group compared with those in the TE group at week 9 (*p* = 0.022, *d* = 0.967). Significant time effects were detected in proprioception thresholds of knee flexion (*p* < 0.001 η2_p_ = 0.540), ankle dorsiflexion (*p* < 0.001 η2_p_ = 0.446), ankle inversion (*p* = 0.048, η2_p_ = 0.148) and eversion (*p* = 0.008, η2_p_ = 0.246).

**TABLE 4:** Proprioception thresholds of the more affected knee joint, ankle sagittal plane, and ankle frontal plane at weeks 0 and 9.

Proprioception threshold (°)		TCHHC + TE (*n* = 13)	TE (*n* = 14)	Time		Group		Time^a^ group		Post hoc	
				p	η^2^_p_	p	η^2^_p_	p	η^2^_p_	p	*d*
Knee flexion	Week 0	3.29 ± 1.57	2.34 ± 1.04	<0.001	0.540	0.095	0.108	0.296	0.043	--	--
	Week 9	1.76 ± 0.51	1.46 ± 0.69							--	--
Knee extension	Week 0	4.29 ± 1.72	3.31 ± 1.64	--	--	--	--	0.018	0.203	^a^ ^1^ < 0.001,^a^ ^2^ = 0.007	^a^ ^1^ = 1.870,^a^ ^2^ = 0.927
	Week 9	1.32 ± 0.31^a^ ^1^ ^b^	1.99 ± 0.93^a^ ^2^							^b^ = 0.022	^b^ = 0.967
Ankle plantarflexion	Week 0	4.40 ± 1.29	3.53 ± 1.22	--	--	--	--	0.010	0.235	^a^ ^1^ < 0.001,^a^ ^2^ < 0.001	^a^ ^1^ = 2.434,^a^ ^2^ = 1.352
	Week 9	1.68 ± 0.61^a^ ^1^	2.10 ± 0.65^a^ ^2^							--	--
Ankle dorsiflexion	Week 0	3.10 ± 1.13	3.22 ± 1.26	<0.001	0.446	0.479	0.020	0.674	0.007	--	--
	Week 9	1.95 ± 0.70	2.27 ± 0.80							--	--
Ankle inversion	Week 0	5.72 ± 2.44	6.04 ± 3.65	0.048	0.148	0.670	0.007	0.921	0.000	--	--
	Week 9	4.54 ± 2.50	4.97 ± 1.67							--	--
Ankle eversion	Week 0	5.51 ± 2.58	5.71 ± 2.93	0.008	0.246	0.268	0.049	0.198	0.065	--	--
	Week 9	3.51 ± 1.21	4.98 ± 1.99							--	--

^a^ Denotes significant different between weeks 0 and 9 in each group.

^b^ Denotes significant difference between the two groups at week 9.

TCHHC: traditional chinese herb hot compress.

TE: therapeutic exercise.

The times of TUG and 20-m walk tests were presented in Table 5. Significant interactions were detected in the TUG (*p* = 0.046, η2_p_ = 0.150) and 20-m walk (*p* = 0.008, η2_p_ = 0.249) tests. Post hoc comparisons showed that compared with week 0, the times of the TUG and 20-m walk tests of both groups (TCHHC + TE: *p* < 0.001, *d* = 4.420, *p* < 0.001, *d* = 3.400; TE: *p* < 0.001, *d* = 3.113; *p* < 0.001, *d* = 2.543) decrease at week 9. The times of the TUG and 20-m walk tests was lower in the TCHHC + TE group compared with those in the TE group at week 9 (*p* = 0.022, *d* = 0.957; *p* = 0.044, *d* = 0.820).

**TABLE 5:** The times of TUG and 20-m walk tests at weeks 0 and 9.

Variables		TCHHC + TE (*n* = 13)	TE (*n* = 14)	Time		Group		Time^a^ group		Post hoc	
				p	η^2^_p_	p	η^2^_p_	p	η^2^_p_	p	*d*
TUG (s)	Week 0	11.67 ± 1.07	11.48 ± 1.14	--	--	--	--	0.046	0.150	^a^ ^1^ < 0.001,^a^ ^2^ < 0.001	^a^ ^1^ = 4.420,^a^ ^2^ = 3.113
	Week 9	7.41 ± 0.80^a^ ^1^ ^b^	8.23 ± 0.91^a^ ^2^ ^b^							^b^ = 0.022	^b^ = 0.957
20-m walk (s)	Week 0	16.61 ± 1.95	15.73 ± 1.58	--	--	--	--	0.008	0.249	^a^ ^1^ < 0.001,^a^ ^2^ < 0.001	^a^ ^1^ = 3.400,^a^ ^2^ = 2.543
	Week 9	10.83 ± 1.18^a^ ^1^ ^b^	11.90 ± 1.42^a^ ^2^ ^b^							^b^ = 0.044	^b^ = 0.820

^a^ Denotes significant different between weeks 0 and 9 in each group.

^b^ Denotes significant difference between the two groups at week 9.

TCHHC: traditional chinese herb hot compress.

TE: therapeutic exercise.

## 4 Discussion

This study investigated the effects of an 8-week TCHHC + TE on pain, proprioception, and functional performance among older adults with KOA. The results supported our hypothesis. Compared with TE, TCHHC + TE was superior in relieving pain, recovering proprioception, and improving functional performance among older adults with KOA.

At week 9, pain scores decreased in both groups, and the decrease was more significant in the TCHHC + TE group than in the TE group. The effects of TE on pain relief have been well documented (Juhl et al., 2014; Zeng et al., 2021). Resistance training increases the muscle strength of the lower extremity, improved the stability of knee joints, and reduces the wear between articular cartilage to relieve pain (Chen H. et al., 2019). Joint mobilization reduces the excitability of nerves to relieve pain (Saunders et al., 2005). Our results showed that TCHHC + TE significantly relieved pain among older adults with KOA compared to TE. Similar results were reported, supporting that the addition of TCHHC to TE enhanced the relief of knee pain (Chen S. et al., 2019). There are several possible mechanisms for TCHHC + TE’s superior effects for pain relief, as follows. 1. Cao (Cao et al., 2016) et al. reported that the herbs have been demonstrated to be effective at promoting absorption of inflammatory substances and eliminating swelling by increasing blood circulation in clinical observation. In this study, some of the components of the herbs are effective in relieving pain, e.g., peony root reduces the expression level of substance P, which is the most important element in pain perception (Wang et al., 2013). Prepared sichuan black and sichuan ox knee reduces the level of pro-inflammatory cytokines (TNF-α, IL-1β, IL-6, and IL-17A) in serum, increases the viscosity of intra-articular knee joint fluid, and eliminates swelling and promotes blood circulation (Weng et al., 2014; Li et al., 2017; Zhang et al., 2019a). Miyajima (Miyajima et al., 2013) et al. point out that hot compress increases the temperature of local tissues and reduces the excitability of afferent nerves, and helps control swelling and reduce pain. TCHHC allow herbs to penetrate the skin barrier through a warming effect, immediately reaching and acting on the affected areas. TCHHC can regulate the imbalance of the levels of anti-inflammatory factors (TGF-β1, IL-13), pro-inflammatory cytokines (IL-1β, IL-6), and pain mediators (PGE2, 5-HT), thus relieving pain (Orita et al., 2011). 2. TE facilitates TCHHC’s pain-relieving effects, by enhancing TE enhances blood and lymphatic fluid circulation, as well as promoting local vasodilation (Guo et al., 2021); thus, it increases the absorption of herbs into the skin and promotes anti-inflammatory and pain-relieving effects.

At week 9, the proprioception thresholds of the knee extension and ankle plantar flexion decreased significantly in both groups, and the decrease was more significant in the TCHHC + TE group than in the TE group. Previous studies showed that TE has a positive effect on the proprioception of knee and ankle joints (Shen et al., 2021), which is consistent with our study. As the most important proprioceptor within the knee joint, the muscle spindles are stimulated by muscle lengthening, speed, or acceleration (Orita et al., 2011).TE releases and stretches the tensed muscles around the knee joint, coordinates the contraction ability between muscles, and enhances muscle spindle sensitivity to improve proprioception among older adults with KOA (Fransen et al., 2002). There are several possible reasons why TCHHC + TE had a superior effect on proprioception, as follows. 1. In this study, some of the components of the herbs have been proven to recover proprioception, such as cassia twig, mulberry parasitic, and antler gum prevent the loss of proteoglycans and accelerate the proliferation of chondrocytes to protect joint cartilage cells and improve their metabolism (Xu and Liu, 2004; Fan et al., 2012), reduce the damage to mechanoreceptors, promote neuromuscular control, and improve proprioception (Chen et al., 2016). Nagashima et al. (Nagashima et al., 2006) and Rusminingsih et al. (Rusminingsih et al., 2020) pointed out that hot compresses applied to the knee joint to dilate blood vessels, increases the excitability of motor neurons and recruits many motor neurons to participate in activities (Levine, 2007), facilitates sensory input. 2. TCHHC enhanced TE’s effects on proprioception. By reducing the excitability of nerve endings and eliminating swelling, thereby allowing the adhesions and atrophy of the knee joint and its surrounding soft tissues to recover as much as possible (Zhang et al., 2015), which facilitates the sensitivity of muscle spindles (Wang et al., 2010; SH, 2012).

At week 9, the times of TUG and 20-m walk decreased significantly in both groups, and the decrease was more significant in the TCHHC + TE group than in the TE group. TCHHC + TE is more effective in improving functional performance, which is consistent with a previous study (Song, 2022). Another study has shown that herbs are used to enhance muscle strength and function performance (Sellami et al., 2018). The level of magnesium (Mg) (Zeng et al., 2015) and calcium (Ca^+^) (Li et al., 2016) in the serum are significantly decreased among older adults with KOA, which reduces their functional performance, increases the risk of falls, and accelerates the deterioration of KOA (Veronese et al., 2017; Heffernan et al., 2020). Some of the Traditional Chinese Herbs, e.g., paeony root, ephedra root, atractylodes macrocephala reduce pro-inflammatory cytokines such as TNF- *α* and interleukins, inhibit the production of matrix metalloproteinases (Yuan et al., 2015; Malemud, 2017), thereby improve the level of Mg and Ca^+^ in serum (Gunn et al., 2013; Zhang et al., 2019a). In addition, parsnip and anemarrhena asphodeloides reduce the expression of fibroblast-like synoviocytes and receptor activator for NF-kB ligand, inhibit the activation of osteoclasts, and reduce the destruction of cartilage, bone, and tendon (Yuan et al., 2015). Previous studies have pointed out that hot compress promotes blood circulation, activates the motor cortex, and facilitates the recovery of adhesions and atrophy of the muscles and soft tissues around the knee joint (Zhang et al., 2015). Therefore, TCHHC is more effective in improving functional performance by expands capillaries, promotes blood circulation, and releases muscle tension (Savaş et al., 2019). The combination of TE facilitates herb penetration in the affected area, reduces stress-related muscle tension, improves lower extremity coordination, and enhances muscle strength to improve functional performance (Guo et al., 2021).

This study has several limitations. First of all, the effects of the TCHHC are possibly attributed to Chinese herbs or hot compress, further studies are recommended to further investigate their specific effects. Second, there was no follow-up after the 8-week intervention, and it was impossible to determine how long the effects would last.

## 5 Conclusion

Compared with TE, the 8-week TCHHC + TE was superior in relieving pain, recovering proprioception, and improving functional performance among older adults with KOA. It is recommended that TCHHC should be adopted prior to TE to enhance the effects of KOA rehabilitation.

## Data Availability

The datasets presented in this study can be found in online repositories. The names of the repository/repositories and accession number(s) can be found below: DOI:10.57760/sciencedb.02151.
